# Strong Phylogeographic Structure in a Millipede Indicates Pleistocene Vicariance between Populations on Banded Iron Formations in Semi-Arid Australia

**DOI:** 10.1371/journal.pone.0093038

**Published:** 2014-03-24

**Authors:** Heidi Nistelberger, Margaret Byrne, David Coates, J. Dale Roberts

**Affiliations:** 1 School of Animal Biology, The University of Western Australia, Crawley, Western Australia, Australia; 2 Science Division, Department of Parks and Wildlife, Bentley Delivery Centre, Bentley, Western Australia, Australia; State Natural History Museum, Germany

## Abstract

The Yilgarn Banded Iron Formations of Western Australia are topographical features that behave as terrestrial islands within the otherwise flat, semi-arid landscape. The formations are characterised by a high number of endemic species, some of which are distributed across multiple formations without inhabiting the intervening landscape. These species provide an ideal context for phylogeographic analysis, to investigate patterns of genetic variation at both spatial and temporal scales. We examined genetic variation in the spirostreptid millipede, *Atelomastix bamfordi*, found on five of these Banded Iron Formations at two mitochondrial loci and 11 microsatellite loci. Strong phylogeographic structuring indicated the five populations became isolated during the Pleistocene, a period of intensifying aridity in this landscape, when it appears populations have been restricted to pockets of moist habitat provided by the formations. The pattern of reciprocal monophyly identified within the mtDNA and strong differentiation within the nuclear microsatellite data highlight the evolutionary significance of these divergent populations and we suggest the degree of differentiation warrants designation of each as a conservation unit.

## Introduction

Islands provide a unique context for studying the evolutionary processes that drive species distributions. They are natural laboratories, allowing examination of processes of speciation, and patterns of colonisation, persistence, dispersal and extinction [1]. The genetic consequences of island distributions are well documented by phylogeographic and population genetic studies that infer historical patterns of natural and sexual selection, drift and their interactions with historical and ongoing dispersal [2]–[5]. The study of terrestrial or ‘habitat’ islands appears to offer a different challenge to oceanic islands, as the matrix between islands can be more permeable than water barriers, resulting in potentially complex spatial and temporal patterns of genetic structure [6], [7]. The most conspicuous and well-studied terrestrial islands include mountain Ranges that form ‘sky islands’, such as the tepui summits of South America, the Western Ghats of southern India and the Madrean and Rocky Mountain sky island systems of central and northern America [6], [8]–[12]. Here, ‘island species’ occupy disjunct populations on either mountain tops or the lowland valleys separating mountains. Other, less conspicuous topographic habitat islands are found throughout the world and include granite outcrops, inselbergs and Banded Iron Formations (BIFs), many of which display high endemism. Although topographically less dramatic, these provide equally compelling bases for phylogeographic study [13]–[17].

Inferring the evolutionary history of terrestrial island endemics can be difficult given that isolation is not always absolute, either spatially and temporally. Dispersal events can occur across valleys and lowlands for mountain-top endemics or over mountains for valley floor endemics, depending on the vagility of the organism and their environmental requirements [6], [7]. Despite this, phylogeographic patterns, although potentially complex, may reveal the degree of connectivity or isolation experienced by island populations.

Restricted gene flow between island populations over long periods of time, will result in substantial genetic divergence due to genetic drift and/or, local adaptation [18]. Given sufficient time, populations may show patterns of reciprocal monophyly, where all individuals can be traced to a common ancestor within that population [19], [20]. Recurrent migration and gene flow between islands on the other hand, has a homogenising effect on genetic structure unless there is strong selection favouring local genotypes on each island. For example, populations of yellow bellied marmots, restricted to the Great Basin mountain-tops of North America, were believed to have been marooned on the rocky outcrops of mountain tops following habitat contraction during the Pleistocene [21]. A pattern of isolation by distance and high genetic diversity showed that occasional dispersal events across the arid scrublands of the Basin have been an important process connecting mountain-top populations [21]. A contrasting pattern was found in the tree *Eucalyptus caesia*, endemic to granite outcrop ‘islands’ of Western Australia. This species has high levels of genetic divergence with little geographic pattern to the distribution of chloroplast haplotypes, reflecting long-term isolation and persistence without connectivity and emphasising the role of genetic drift in this landscape [22].

The Yilgarn Banded Iron Formations (BIFs) of south-western Australia are ancient, island-like rock outcrops that provide an ideal system in which to investigate island population dynamics. They border the transitional rainfall zone, a biogeographic region separating the high rainfall forest region from the arid interior and are characterised by high species endemism and richness [14], [23]. BIFs vary widely in height (highest point approx. 700 m above sea level), shape and distance from one another and are comprised of banded iron talus slopes and areas of weathered duricrust [24]. These topographically complex formations occur in an otherwise flat landscape that is dominated by clay, silt and sand plains. The region has been geologically stable, un-glaciated and above sea level since the Permian (250 mya) [25]–[27]. Nonetheless there have been substantial changes in climate and vegetation communities over this time. At the beginning of the Cenozoic (65 mya) the region was warm, humid and dominated by temperate rainforest [28]. Over time, the landscape became increasingly arid, beginning approximately 11 mya in the mid-Miocene, shown by the absence of regular flows in paleo-drainage systems [28]. Another aridification event occurred 7 mya in the late Miocene with several more throughout the Pliocene, shown by changes in vegetation structure in palynological deposits to more arid-adapted forms [28], [29]. By the early Pleistocene (approx. 2.6 mya), the modern climate had more or less become established but fluctuations throughout this period continued, associated with successive glacial and interglacial periods, the latter with intensifying arid conditions [28].

Historically, the altitudinal relief of BIFs and their complex structure, including deep valleys and shaded gullies as well as exposed outcrops and talus slopes [24], would have provided an abundance of niche habitats that allowed for the persistence of both mesic and xeric-adapted species during changing climates [30]. Whilst this may account for the high species diversity found on the BIFs today, the high endemism [14], [23], [31] also suggests long periods of isolation, allowing for speciation and the development and persistence of a unique flora and fauna. The Yilgarn BIF endemics consist of species that are localised (found on one BIF), or regional (located on several BIFs but not in the intervening landscape) [23]. Regional endemics provide an opportunity to study phylogeographic patterns, to determine what processes have caused current distributions, and historically, how diversity has evolved and accumulated. The floral assemblages of the Yilgarn BIFs are well known, with many examples of regional endemics [23]. Although less is known of the faunal assemblages, taxa that exhibit short-range endemism consistent with limited dispersal capacity, such as millipedes, mygalomorph spiders, land snails and troglofauna [32]–[34], may also exhibit patterns of regional endemism, persisting in micro-habitats provided by the BIF.

We investigated phylogeographic pattern in the spirostreptid millipede *Atelomastix bamfordi*, known to occur on five BIFs on the Yilgarn Plateau in Western Australia. Millipedes lack the waxy cuticle that protects other arthropods from desiccation and are reliant on moist environments for survival [32], [35]. *Atelomastix* from the family Iulomorphidae, is a predominantly south-western Australian genus with many species distributed across the wetter parts of the southern and south-western coastline and mesic inland Ranges, including the Stirling and Porongorup Ranges [36], [37]. Almost all species have restricted distributions and have been characterised as short-range endemics (geographic range <10000 km^2^) [32]. *Atelomastix bamfordi* is the most easterly occurring member of this genus. It inhabits moist habitats provided by BIFs, such as the base of rocky slopes, under large rocks and under dense leaf litter. The life cycle is probably annual with adult millipedes dying prior to, or during the harsh summer months (pers. comm. M Harvey). The species has been reported as abundant on the large Koolyanobbing Range [33] but appears scarcer on the drier BIFs to the north and east. Increased exploration and mining of iron ore within the region [23] has resulted in the need for a delicate balance between mining activities and conservation of BIF endemic species. Understanding the patterns and evolution of diversity in these sites may guide two key conservation issues: i) the viability and conservation of highly endemic species ii) the conservation of landscape features which maximise the chances of maintaining patterns of dispersal, gene flow and local adaptation that have shaped the history of extant diversity and continue to be major factors in the persistence of this diversity.

Although unconnected today, BIF populations of *A. bamfordi* may have repeatedly come into contact through population expansion associated with historical climate change in this landscape. If this were the case, we would expect periods of gene exchange between neighbouring BIF populations to result in a pattern of isolation-by-distance, where proximate populations are more genetically related, or, if gene exchange has been extensive, to genetic homogeneity of populations [38]. Alternately, the current distribution may be due to contraction of a formerly widespread ancestor followed by long-term isolation and persistence on BIF islands. If this were the case, we would expect to see signatures of deep genetic divergence amongst BIF populations but no clear pattern of isolation by distance. Haplotypes would be conserved and there may be evidence of reciprocal monophyly, if sufficient time for lineage sorting has occurred [39]. This is the first phylogeographic study to examine patterns of population level divergence in a regional BIF endemic species from this important, biodiverse region.

We evaluated the genetic variation present in *A. bamfordi* to investigate evolutionary relationships and patterns of genetic diversity and structure across BIF populations. Our goals were:

1. To determine patterns of genetic differentiation among the five known BIF populations and to use those data to infer the impact of historical processes on the distribution of millipedes across the landscape.
2. To assess genetic diversity and structure to determine how limited dispersal capabilities affects structure within BIF millipede populations.
3. To use patterns of genetic differentiation, both within and among BIFs to inform conservation and management of this species.

## Materials and Methods

### Sampling and DNA extraction

*A. bamfordi* had been recorded from four of the Yilgarn BIFs: Koolyanobbing (KOO), Windarling (WIN), Mt Jackson (MTJ) and the Die Hardy Range (DH) (pers. comm. R. Teale) [33]. This study extended the known distribution to include the Helena Aurora (HA) Range. Four more BIFs in the region (Mt Dimer, Mt Correll, Hunt Range and Mt Manning) were also searched but no specimens were found. Specimens from the Windarling and Koolyanobbing Ranges, preserved in 100% ethanol were available from the Western Australian Museum's Department of Terrestrial Zoology collection (Table S1). For the remaining BIFs (DH, MTJ and HA) sampling was spread across the geographic extent of each BIF as far as was practical to avoid sampling closely related individuals. For some BIFs this was not possible as millipedes were either not found and/or there was no suitable habitat. The same search procedure was applied to all BIFs, so we believe it is reasonable to assume that the specimens collected reflect the genetic diversity contained within each site. The five BIFs vary in size, topographic complexity and orientation. Koolyanobbing is large, approximately 12 km in length and reaching heights of 500 m. This BIF is topographically complex and contains many sites with habitat suitable for millipedes. Helena Aurora is also large (∼12 km) and elevated (600–700 m height), but is comprised of more rounded hills with gentle slopes. Millipedes here were associated with drainage lines coming off the peaks. The Die Hardy Range is large and X-shaped, a topology providing many shaded slopes that supported habitat suitable for millipedes. The two smaller Ranges Windarling and Mt Jackson, have a narrow, east to west orientation and are therefore subject to an abundance of northern sunlight. These smaller Ranges did not possess as many water-gaining sites and millipedes were scarcer. Invertebrate surveys conducted on the intervening ‘flats’ between the Yilgarn BIF have not recorded *A. bamfordi* (pers. comm. R. Teale). We also searched any water-gaining or densely vegetated sites between BIFs and although these recovered other millipedes from the genus *Antichiropus*, *A. bamfordi* was not found. Thus, to the best of our knowledge, *A. bamfordi* populations are restricted to BIF habitat and are not presently connected by populations in intervening habitat. The field study did not involve any endangered or protected species and none of the field sites from which we sampled (i.e. excluding KOO and WIN) required access permission, with the exception of Mt Jackson (permission required from Cliffs Natural Resources) and Mt Manning (Regulation 4 permit required from the Western Australian Department of Parks and Wildlife (DPaW)). A license to take fauna for scientific purposes was obtained prior to sampling from DPaW. GPS coordinates of the sampling locations are provided in Table S1.

In total, 77 individuals from five BIFs were sequenced at two mitochondrial regions (77 at 16 S, 75 at CO1) and genotyped at 11 nuclear microsatellite loci. DNA was extracted from the legs or from the crushed heads of juvenile specimens stored in 100% ethanol using a Qiagen DNeasy Kit, or a salt-based DNA extraction protocol [40]. Elutions were performed in 100 μl or 50 μl of TE buffer respectively. Legs and head tissue were homogenized in the lysis buffer prior to addition of proteinase K using mini micropestles (Geneworks).

### Mitochondrial DNA amplification and sequencing

We sequenced two partial regions of the mitochondria, the Cytochrome Oxidase Subunit 1 (CO1) and the large Ribosomal Subunit 16 S. For amplification of CO1 we used the Folmer primers LCO1490 (5′GGTCAACAAATCATAAAGATATTGG3′) and HCO2198 (5′TAAACTTCAGGGTGACCAAAAAATCA3′) [41] and for 16 S, the ‘universal’ primers 16Sar (5′CGCCTGTTTTTCAAAAACAT3′) and 16Sbr (5′CCGGTTTGAACTCAGATCATGT3′) [42]. We also amplified two nuclear gene regions, the Internal Transcribed Spacer 1 (ITS-1) [43] and the 18S rRNA gene [44]. ITS-1 sequenced products showed evidence of site heterogeneity and were excluded from use, and 18 S sequences were monomorphic. Amplifications were performed in 25 μl reactions containing 1× PCR buffer, 3 mM MgCl_2_, 200 μM dNTP's, 0.4 μM of each forward and reverse primer, 0.25 μg of Bovine Serum Albumin (Fisher Biotech), 0.3 U of Platinum Taq DNA Polymerase (Invitrogen) and approximately 20 ng/μl DNA. The thermal cycler profile for both CO1 and 16 S involved an initial denaturation at 94°C for 3 min, followed by 35 cycles of 94°C for 30 sec, 46°C for 30 sec and 72°C for 30 sec followed by a final extension at 72°C for 2 min. PCR products were purified using an UltraClean PCR Cleanup kit (Mo-Bio technologies, Geneworks) and the cleaned product was quantified using a Nanodrop (Nanodrop Technologies). Sequencing reactions (1/8) were carried out in 10 μl volumes containing 1 μl of Big Dye Terminator, 1.5 μl of 5× sequencing buffer, 1 μl of 3.2 pmol primer and approximately 40 ng of purified PCR product. Thermal cycling conditions for sequencing reactions involved an initial denaturation at 96°C for 2 min, followed by 25 cycles of 96°C for 10 sec, 46°C for 5 sec and 60°C for 4 min. Sequenced products were cleaned prior to electrophoresis using a standard ethanol precipitation method (Applied Biosystems). Products were sequenced on an Applied Biosystems 3730 capillary sequencer (Murdoch University).

### Mitochondrial DNA analysis

Sequence data were aligned using Clustal W in the program BioEdit [26] and finalised by eye with all mutations checked against raw sequence data. We used AMOVA, implemented in Arlequin v. 3.5 [45] to determine the structuring of mitochondrial DNA diversity amongst BIF populations using the number of pairwise base-pair differences and tested for significance with 10000 permutations. Bayesian analysis was used to visualise genetic relationships and to estimate coalescence times of the populations in BEAST v. 1.7.5 [46]. Data were partitioned into the CO1 and 16 S datasets, enforcing monophyly for all *A. bamfordi* samples, with a SRD06 codon-partitioned model of sequence evolution applied to the CO1 dataset and an HKY+G model to the 16 S partition. Due to an absence of fossil data and geological calibration points, estimates of population coalescence times were generated by applying the average arthropod mutation rate of 2.3% per million years to the partitioned data set [47]. We applied a strict clock to both partitions and used a Coalescent-Constant Size tree prior for four independent runs of 40 million generations, sampling every 1000 generations. Log files were combined in Log Combiner and the first 25% discarded as burnin. Visual inspection of the traces and high ESS values (>200) confirmed stationarity had been reached in Tracer (BEAST package). Tree files were combined in LogCombiner and the final file annotated in TreeAnnotater (BEAST package).

To distinguish between incomplete lineage sorting and migration for the two populations that were paraphyletic (see results), we performed nested model likelihood ratio tests of various isolation versus migration models in ‘L mode’ in the program IMa2 [48]. IMa2 uses Markov chain Monte Carlo simulations of gene genealogies to estimate divergence times of populations and effective population sizes of extant and ancestral populations [49]. To assess whether migration or drift was responsible for the paraphyly observed in the two populations we performed a run on the two populations in ‘M mode’ using the following command line priors: q200, t10, m1, b100000, l1000000, hfg, hn6, ha0.96, hb0.9. Convergence was assessed by monitoring ESS values (all >20 000), chain swap rates (>80%) and running the same command line four times independently using different seed values to ensure consistency of results. The output was then used to test the likelihood of 24 different migration/isolation models outlined in Hey and Nielsen [50] using the following command line: q200, t10, m1, r0, c2 b100000 l1000000. Likelihood ratio tests were used to reject models that were significantly worse than the full model, and the remaining models were compared using the AIC criterion (AIC = −2(log(P)+2K)) to identify the model of best fit, where K is the number of parameters in the statistical model and L is the maximised value of the likelihood function [51].

To assess the historical stability of BIF population sizes we performed tests of neutrality that can also serve as a measure of demographic expansion or contraction. Estimates of Tajima's D were calculated in Arlequin. Diversity statistics including haplotype diversity, nucleotide diversity (Pi), and nucleotide divergence (Jukes and Cantor) between populations were calculated in DnaSP v. 5.10 [52].

To test for isolation by distance within BIF populations using the combined sequence data, we conducted mantel tests in R using the Vegan package [53]. Permutations were performed 10000 times on matrices of the number of pairwise sequence differences and geographic distance. The R statistic is based on Pearson's product-moment correlation coefficient. We plotted the number of pairwise differences on geographic distance and established a linear relationship meaning no transformations of the data were required.

### Nuclear microsatellite genotyping and data analysis

Genotyping of the 11 microsatellite markers was carried out according to Nistelberger et al. [54]. We checked all pairwise combinations of loci for linkage disequilibrium by performing exact tests in Genepop v.1.2 [55] and applying a sequential Bonferroni correction to determine significance [56]. There was no significant association among loci following sequential Bonferroni correction. Traditional F statistics were of limited use in our study due to the likelihood that our BIF ‘populations’ do not represent true panmictic populations given the distances over which individuals were sampled and the limited dispersal capabilities of *A. bamfordi*. This was confirmed with all populations showing significant deviation from Hardy-Weinberg equilibrium, with the exception of Windarling, where individuals had been sampled in close proximity to one another. Rarefied allelic richness provided a better estimator of genetic diversity, given the sampling, and was calculated in HP-Rare v 1.0 [57]. The partitioning of genetic diversity amongst BIF populations was assessed using an Analysis of Molecular Variance (AMOVA) implemented in Arlequin with significance determined with 10000 permutations. An analysis of genetic structure across the five BIF populations was assessed using a Bayesian clustering algorithm implemented in TESS ver. 2.3 [58]. This program assumes proximate interactions between individuals with spatial information prescribed at the individual level. We ran TESS with no admixture, owing to the limited dispersal capabilities of millipedes and performed 100 iterations of K values between 1 and 7, with a burnin length of 20000, 100000 sweeps and a spatial interaction parameter of 0.8. Lowest DIC values for each K value were plotted in order to determine the optimal K value. The results were then used in the downstream program CLUMPP [59] which aligns cluster assignment across the replicate analyses and ensures the correct assignment of K. Structure output graphics were created using DISTRUCT [60].

To further reconstruct the phylogenetic relationships of populations using microsatellite data we generated Cavalli-Sforza chord distances (D_C_) [61] between populations and determined support using 100 bootstrap replications in the program Populations 1.2.30 [62]. This geometric distance measure does not make any biological assumptions about the data and can be more useful for phylogenetic inference than estimates that use stepwise mutation models when small population sizes are involved [61], [63]. Distances were imported into TreeView for visualisation [64]. To assess isolation by distance within BIF populations using microsatellite data we correlated pairwise population matrices of genetic distance and geographic distance and tested for significance using a Mantel test with 9999 permutations in the program IBDWS v 3.23 [65].

## Results

### Genetic variation

#### Mitochondrial DNA data

Of the two mitochondrial regions amplified, CO1 was the most variable, with 45 haplotypes identified from the 75 individuals sequenced (two WIN samples did not amplify at CO1). The variation was composed of 54 transitions, eight transversions and two multistate substitutions. The 16 S region yielded 24 haplotypes from the 77 individuals sequenced and included 21 transitions, one transversion and one single base pair indel. Combining the two regions resulted in a total, aligned sequence length of 1114 bp and 48 haplotypes. The most prevalent haplotype (Hap 47 WIN) had a frequency of 23%, followed by Hap 35 (MTJ) and Hap 39 (HA) with frequencies of 8%, and Hap 33 (MTJ) and Hap 40 (HA) with 6%. The remaining haplotypes were only found in one or two individuals. Measures of genetic diversity were consistently lowest in the Windarling population and highest in Koolyanobbing. Haplotype diversity ranged from 0.295 (WIN) to 0.986 (KOO) with an average of 0.794. Nucleotide diversity ranged from 0.06% (WIN) to 8.5% (KOO) with an average of 4%. No populations departed from the neutral model of evolution except for Windarling (D = −1.775) (Table 1). Measures of diversity in Windarling are likely to be biased due to restricted sampling. Millipedes had been previously collected from two, closely located sites on this BIF (<260 m apart), and although noted to be rare along the length of the Range (pers. comm. R. Teale), further sampling was not possible due to restricted access associated with mining activity.

**Table 1:** Diversity statistics for the five *Atelomastix bamfordi* BIF populations for mitochondrial DNA data and nuclear microsatellite data (bold), standard error in parentheses: N, number of individuals; # haps, number of haplotypes; HD, haplotype diversity; Pi, nucleotide diversity; D, Tajima's D; n, average number of individuals genotyped; AR∧, rarefied allelic richness; * P value<0.05.

	Population		#				Haplotype	Genbank Accession #			
Population	abbrev.	N	haps	HD	Pi	D	numbers	CO1	16 S	n	AR∧
Die Hardy Range	DH	16	14	0.983 (0.002)	0.0050 (0.000)	−0.569	19–32	KC689871–KC689883	KC689837–KC689841	**13.7**	**2.62 (0.34)**
Helena Aurora Range	HA	14	8	0.890 (0.004)	0.0042 (0.000)	−0.733	38–45	KC689889–KC689894	KC689845–KC689851	**13.9**	**1.89 (0.28)**
Koolyanobbing Range	KOO	21	18	0.986 (0.001)	0.0085 (0.000)	−0.316	1–18	KC689853–KC689870	KC689829–KC689836	**21.7**	**3.28 (0.40)**
Mt Jackson	MTJ	11	5	0.818 (0.008)	0.0017 (0.000)	−0.234	33–37	KC689884–KC689888	KC689842–KC689844	**9.6**	**2.57 (0.36)**
Windarling Range	WIN	13	3	0.295 (0.012)	0.0006 (0.000)	−1.775*	46–48	KC689895–KC689897	KC689852	**14**	**1.97 (0.27)**

**Figure 1:**
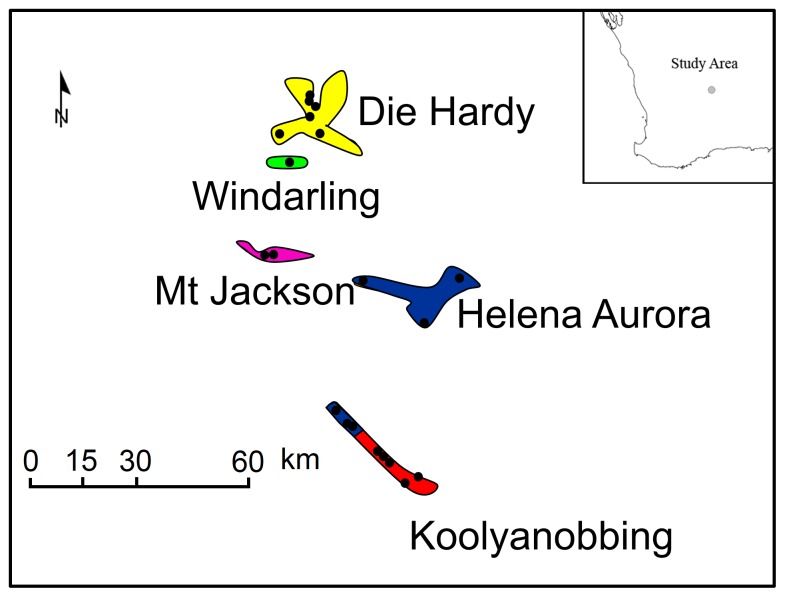
Schematic representation of the five *Atelomastix bamfordi* Banded Iron Formation populations, coloured according to the five genetic clades identified with mtDNA analysis. Black circles indicate sample sites. Study area shown in inset.

There was a large and highly significant degree of genetic structuring of the mitochondrial genetic diversity, with most variation (AMOVA) occurring among BIF populations (71.56%) rather than within (28.4%). The greatest genetic divergence occurred between the Mt Jackson and Helena Aurora populations (approx. 40 km apart) and the least between the two most spatially isolated populations, Koolyanobbing and Die Hardy (approx. 100 km apart) (Table 2, Fig. 1).

**Table 2:** Pairwise population nucleotide divergence based on Jukes and Cantor of five *Atelomastix bamfordi* populations using mtDNA data.

	DH	HA	MTJ	WIN	KOO
DH	0				
HA	0.016	0			
MTJ	0.017	0.022	0		
WIN	0.013	0.018	0.019	0	
KOO	0.013	0.014	0.018	0.017	0

*P value<0.005.

#### Nuclear microsatellite data

Microsatellite genetic diversity was low, both within and amongst BIF populations. Rarefied allelic richness ranged from 1.89 (HA) to 3.28 (KOO) with an average of 2.47. There was a highly significant degree of structuring of the microsatellite diversity with the majority of variation (68.11%) maintained within populations, compared to (31.89%) among populations.

### Phylogeographic structure and population genetic structure

**Figure 2:**
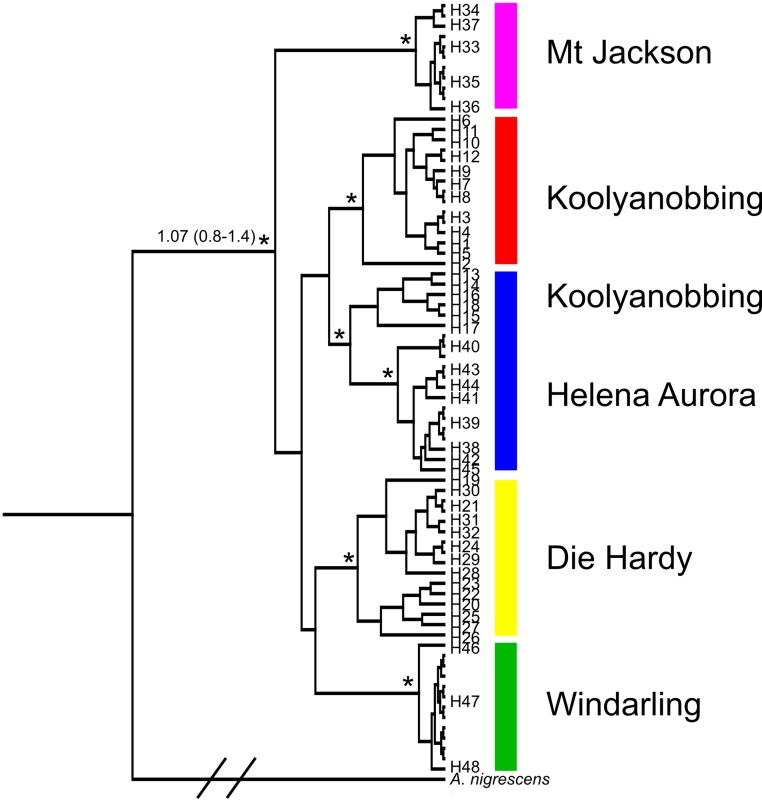
Bayesian cladogram of 75 *Atelomastix bamfordi* individuals based on combined CO1 and 16 S mtDNA data. Clades are designated separate colours as per Fig. 1. Numbers on terminals indicate haplotype number. The * denotes major nodes with high support (posterior probability>0.85) and the numbered node indicates divergence estimate in millions of years with confidence intervals in brackets. NB the *A. nigrescens* branch has been truncated (//) for viewing purposes.

There was evidence of strong phylogeographic structure within *A. bamfordi*, with minimal sharing of CO1 and 16 S haplotypes amongst individuals within BIF populations and no sharing of haplotypes between populations. Bayesian analysis identified five genetic clades in which populations were reciprocally monophyletic, with the exception of Koolyanobbing and Helena Aurora for which some samples were paraphyletic (Fig. 2). This was due to a subset of six individuals from the north of the Koolyanobbing Range being grouped with samples from Helena Aurora (Fig. 1). Support for the five genetic clades was high (posterior probabilities ≥0.85) although the relationships between the clades was poorly supported. Dating estimates showed coalescence during the Pleistocene, between 0.729 Ma [0.532–0.952] and 1.078 Ma [0.792–1.433], although only the date of 1.078 for the divergence of the Mt Jackson population has high support (Fig. 2).

Isolation and migration analysis in the program IMa2 was used to determine whether the paraphyly between Koolyanobbing and Helena Aurora was due to incomplete lineage sorting (isolation) or gene flow (migration). The three models with the highest AIC support all indicated there was no historical migration between the two populations (Table S2).

Mantel tests of pairwise sequence differences against geographic distance indicated significant isolation by distance within the Koolyanobbing (r = 0.740 p = 0.0001), Mt Jackson (r = 0.225 p = 0.0375) and Helena Aurora (r = 0.399 p = 0.0025) populations.

### Population genetic structure

**Figure 3:**
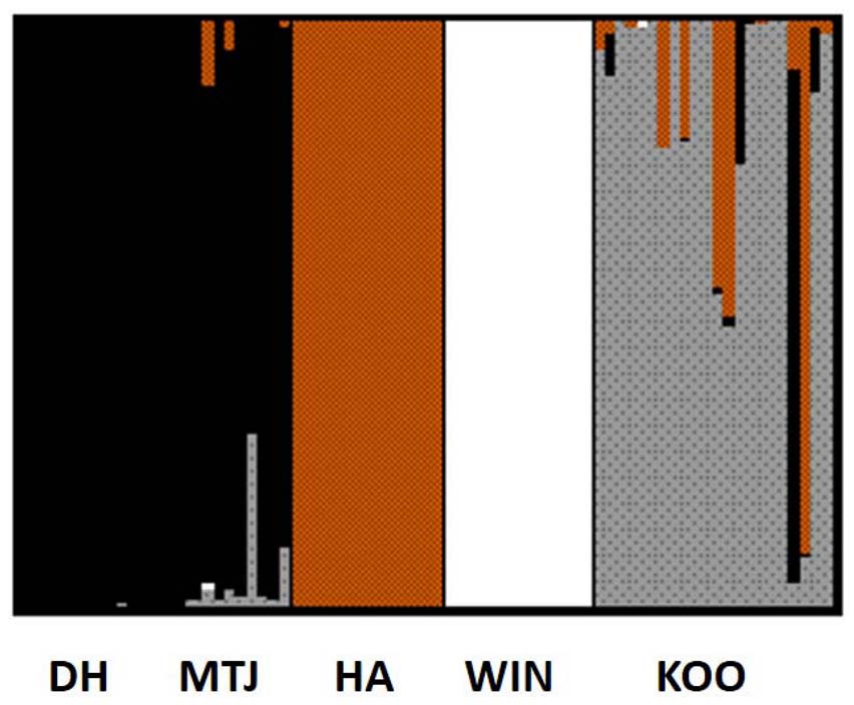
Bayesian population assignment of *Atelomastix bamfordi* individuals generated in TESS for the optimal grouping of K = 4. Each vertical line represents an individual and the colour displays the probability of belonging to each genetic cluster.

**Figure 4:**
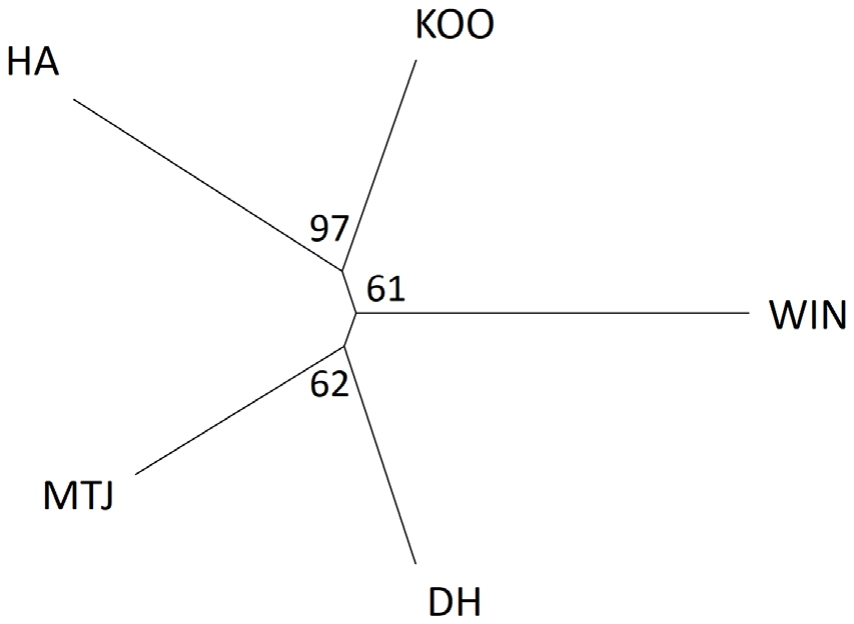
Unrooted neighbour-joining tree of Cavalli-Sforza chord distances between the five BIF populations of *Atelomastix bamfordi*. Distances are based on 11 microsatellite loci with bootstrap support shown at nodes.

Bayesian cluster analysis identified four genetic clusters in the microsatellite data. The first corresponded to the Die Hardy and Mt Jackson populations, and the remaining clusters to each of the three other populations (HA, WIN, KOO) (Fig. 3). Further investigation of structure in the first cluster revealed two more groupings corresponding to the separate BIF populations (DH, MTJ). There was some evidence of admixture in the Mt Jackson and Koolyanobbing populations (Fig. 3). Phylogenetic analysis of relationships between populations using Cavalli-Sforza chord distances showed distinct genetic structure amongst populations using microsatellite data, but with some clustering of Koolyanobbing and Helena Aurora (97% bootstrap support) and Die Hardy and Mt Jackson (62%) (Fig. 4). Two of the five populations, Helena Aurora (r = 0.30 p = 0.004) and Die Hardy (r = 0.32 p = 0.009), showed weak but significant patterns of isolation-by-distance at microsatellite loci following Mantel tests.

## Discussion

### Genetic differentiation of BIF populations

#### Spatial patterns

Phylogeographic analysis of *A. bamfordi* has revealed strong genetic differentiation between BIF populations, with mtDNA genetic lineages corresponding to each of the five BIF populations except for six individuals from Koolyanobbing that were paraphyletic with the Helena Aurora Range due to incomplete lineage sorting. Microsatellite data were broadly consistent with this result, identifying four genetic clusters, the first of which was further broken down into individual populations.

The lack of correlation between genetic divergence and geographic distance, with the two most spatially isolated populations showing the least genetic divergence, suggests there has been little, to no connectivity of BIF populations following their separation. This result was emphasized in the Bayesian analysis which showed high support for genetic clades but poor support for the relationships between clades (Fig. 2). It is also consistent with estimates of sequence neutrality (D) that indicated a lack of demographic expansion and contraction events, with the exception of Windarling, where the result can be explained by close sample proximity. This pattern of differentiation has been observed in other taxa with restricted dispersal capabilities where populations have become isolated as a response to climate change and habitat loss. Like *A. bamfordi*, these studies emphasise the role of genetic drift in driving population differentiation. For example, mitochondrial and nuclear sequence data revealed extraordinarily strong phylogeographic structure in populations of trapdoor spiders (*Moggridgea tingle*) in south-western Australia, often situated just kilometres apart. Molecular dating suggested these populations became isolated in the mesic valleys and uplands of the Porongorup and Stirling Ranges following the onset of aridity in the late Miocene [66]. Like *A. bamfordi*, reciprocally monophyletic lineages across three geographic locations highlighted the reproductive isolation of *M. tingle* populations but the pattern emphasized a more ancient division, with monophyly observed in both mtDNA and the more slowly evolving nuclear genome [66]. Studies of genetic structure in two frog species in south-western Australia, *Geocrinia lutea* and *G. rosea*, also described patterns of extremely low dispersal and long-term isolation between habitat ‘islands’, with divergence attributed to isolation via range changes resulting from climate change rather than geographical barriers [67]. Another striking example of genetic divergence across terrestrial islands occurred in the sky islands of south-eastern Arizona - forested mountain Ranges, separated by low-lying grassland and desert. Here, the jumping spider *Habronattus pugillis* occupies woodland habitat at altitudes above 1400 m but is absent from the lowlands. Using mtDNA, Masta [7] showed that like *A. bamfordi*, smaller populations of *H. pugillis* formed monophyletic clades whereas larger Ranges showed evidence of paraphyly. Like *A. bamfordi*, this was ascribed to incomplete lineage sorting rather than resulting from migration [7]. The isolation of populations was attributed to climate-induced contraction of woodland populations onto disjunct mountain Ranges.

#### Temporal patterns

Molecular dating with the arthropod clock rate has placed the separation of *A. bamfordi* clades at 0.7 to 1.1 million years ago during the Pleistocene. The timing coincides with known increases in aridity in the transitional rainfall zone (TRZ) due to climatic fluctuations associated with Milankovitch cycles during this period [68]. In particular there was a major arid shift in southern Australia corresponding to the middle Pleistocene transition (1250 ka–700 ka), a period of increased severity of glaciation, associated with greater ice volumes, lowered temperatures and increased aridity [69]. Although the TRZ remained un-glaciated, this period of intense climatic instability is expected to have caused the fragmentation of species distributions due to changes in suitable habitat within this region [70]. Although the divergence estimates presented here must be treated with caution given the use of a generalised arthropod mutation rate, the errors associated with this method are lessened given we are working at shallow phylogenetic levels [71], [72].

### BIF population dynamics – historical and contemporary patterns

#### Historical patterns

The process of genetic drift acting independently on each BIF population is reflected in different patterns of genetic divergence and diversity seen in the mtDNA data. The smaller populations, Mt Jackson and Windarling, and the larger population Helena Aurora, which had less suitable millipede habitat, showed lower genetic diversity and higher levels of genetic divergence than the larger populations (KOO and DH). These three BIFs have probably harboured historically small effective population sizes, owing to the limited availability of moist sites, increasing their susceptibility to forces of genetic drift driving divergence [73]. They may also be influenced by different selective forces associated with the more arid conditions present on these BIFs. On the other hand the two large BIF populations, characterised by a greater availability of suitable habitat (KOO and DH), retained genetic diversity and had less genetic divergence, consistent with historically large population sizes [73]. The paraphyly of individuals from Koolyanobbing with those of Helena Aurora further supports the likelihood of a large historical population size at Koolyanobbing, as lineage sorting is still ongoing at this BIF. Over time this signature is likely to erode, leaving the five BIFs reciprocally monophyletic.

#### Contemporary patterns

Rapidly evolving microsatellite markers can provide a more contemporary view of BIF population dynamics. The information we gained from these markers was limited by a lack of Hardy-Weinberg equilibrium within our BIF populations yet we still identified patterns in genetic diversity that appear to support the impact of genetic drift on populations: lower genetic diversity in BIF's likely to have had small effective population sizes (WIN, MTJ and HA). Signals of isolation by distance (IBD) were seen in some of the BIF populations (HA, DH), but these were weak and likely to be heavily influenced by limited sample sizes. The small pairwise distances between samples at Windarling explains the lack of IBD seen in this BIF, but we were surprised to see evidence of IBD in the Helena Aurora and Mt Jackson individuals. Here, we expected limited gene flow to result in a pattern of ‘islands within islands’, where significant isolation has resulted in genetically divergent populations within BIFs that do not correlate with geographic distance. An increased number of samples from more locations would be required to determine whether IBD is really occurring at these spatial scales.

### Conservation implications

The evolutionary significance of geographically isolated genetic lineages has long been recognised and conservation biologists have attempted to formalise this by delineating conservation units within a species such as Evolutionary Significant Units (ESUs) and Management Units (MUs) [74]. Conservation units have been defined at various levels of genetic differentiation within species and are likely to experience limited gene flow or even total reproductive isolation. For example, ESUs have been defined as a population or a group of populations that warrant separate management for conservation because of high genetic and ecological distinctiveness [75], with Mortiz [20] suggesting that ESUs might be characterised by a pattern of reciprocal monophyly amongst populations. Applying this criterion to our mtDNA data, results in the recognition of three BIF populations (DH, MTJ, WIN) as ESUs. However, Paetkau [76] and Crandall et al. [77] highlight the problems inherent in ignoring paraphyletic lineages, such as Koolyanobbing and Helena Aurora, which are often ancestral and maintain high levels of diversity and divergence within themselves. In our case, the paraphyly of Koolyanobbing with Helena Aurora is a result of a slower progression towards monophyly due to ongoing lineage sorting, probably owing to large population sizes. Given the clear evidence for restricted gene flow between these two populations, both could readily be recognised as MUs. Therefore we consider that all five BIF populations warrant designation as conservation units.

Although it is difficult to predict future climate change at such fine scales, broad-scale models for this region over the 21^st^ century indicate increasing temperature, decreasing rainfall and a greater incidence of fire [78]. If these predictions are correct, populations are unlikely to re-connect via suitable intervening habitat in the future, further consolidating their isolation to the remaining moist pockets provided by BIF, where they will continue to be influenced by forces of genetic drift and local selection. Given their dependence on moisture, conservation efforts should focus on protecting areas of dense leaf litter, large outcrops and shaded gullies on each BIF in order to preserve the five genetic lineages identified here. To achieve this, preservation of the height and topographical complexity of BIFs is essential, in order to maintain drainage lines and water-collecting sites. Conservation of these areas is also likely to support populations of other, relict fauna and flora that are adapted to moist environments.

## Conclusion

This is the first phylogeographic study conducted on a species inhabiting multiple Banded Iron Formations in this landscape and provides evidence that the disjunct populations have experienced long-term isolation following their separation during the Pleistocene. The divergence of populations during this period coincides with increases in aridity and emphasizes the role of climatic instability along with topographic features in driving differentiation and ultimately speciation in this landscape. We consider the strong phylogeographic structure and evidence of restricted gene flow between BIF populations warrant the designation of all five populations as distinct conservation units.

## Supporting Information

Table S1 **Specimen information, including spatial coordinates from UTM zone 50 J and haplotype number based on combined CO1 and 16 S mtDNA data.** Samples from Windarling and Kooylanobbing (bold) are from specimens lodged at the Western Australian Museum, registration numbers shown (WAM_T).
(DOCX)

Table S2 **The three, best-fit models determined in IMa2 for tests of migration versus isolation between the paraphyletic populations Koolyanobbing and Helena Aurora.** Results are based on mtDNA variation.
(DOCX)
