# Supplementary material for: Strong Phylogeographic Structure in a Millipede Indicates Pleistocene Vicariance between Populations on Banded Iron Formations in Semi-Arid Australia
Source: PLoS One. 2014 Mar 24;9(3):e93038. doi: 10.1371/journal.pone.0093038 (PMC3963978; doi:10.1371/journal.pone.0093038)
Supplement: Table S1 — Specimen information, including spatial coordinates from UTM zone 50 J and haplotype number based on combined CO1 and 16 S mtDNA data. Samples from Windarling and Kooylanobbing (bold) are from specimens lodged at the Western Australian Museum, registration numbers shown (WAM_T). (DOCX) [file pone.0093038.s001.docx]

| Sample | Population | Easting | Northing | Haplotype no. |
| --- | --- | --- | --- | --- |
| DH23 | Die Hardy | 729291 | 6685360 | Hap21 |
| DH2 | Die Hardy | 725805 | 6684839 | Hap19 |
| DH32 | Die Hardy | 727525 | 6687269 | Hap24 |
| DH33 | Die Hardy | 727525 | 6687269 | Hap25 |
| DH36 | Die Hardy | 727458 | 6687278 | Hap26 |
| DH37 | Die Hardy | 727458 | 6687278 | Hap27 |
| DH40 | Die Hardy | 727411 | 6687282 | Hap28 |
| DH41 | Die Hardy | 727347 | 6687287 | Hap29 |
| DH43 | Die Hardy | 727257 | 6687301 | Hap24 |
| DH46 | Die Hardy | 728060 | 6690423 | Hap30 |
| DH47 | Die Hardy | 728411 | 6690546 | Hap31 |
| DH48 | Die Hardy | 728411 | 6690546 | Hap32 |
| DH6 | Die Hardy | 729215 | 6685314 | Hap20 |
| DH7 | Die Hardy | 729215 | 6685314 | Hap21 |
| DH9 | Die Hardy | 729215 | 6685314 | Hap23 |
| HA36 | Helena Aurora | 760332 | 6639565 | Hap42 |
| HA37 | Helena Aurora | 760332 | 6639565 | Hap43 |
| HA38 | Helena Aurora | 760332 | 6639565 | Hap44 |
| HA41 | Helena Aurora | 760320 | 6639552 | Hap43 |
| HA42 | Helena Aurora | 760270 | 6639470 | Hap45 |
| HNAF11 | Helena Aurora | 752715 | 6637738 | Hap39 |
| HNAF13 | Helena Aurora | 754411 | 6633431 | Hap40 |
| HNAF18 | Helena Aurora | 754419 | 6633434 | Hap40 |
| HNAF19 | Helena Aurora | 754419 | 6633434 | Hap41 |
| HNAF24 | Helena Aurora | 754414 | 6633429 | Hap40 |
| HNAF2 | Helena Aurora | 752978 | 6637869 | Hap38 |
| HNAF3 | Helena Aurora | 752978 | 6637869 | Hap39 |
| HNAF4 | Helena Aurora | 752978 | 6637869 | Hap39 |
| HNAF5 | Helena Aurora | 752978 | 6637869 | Hap39 |
| MTJ39 | Mt Jackson | 718999 | 6650761 | Hap35 |
| MTJ41 | Mt Jackson | 719010 | 6650756 | Hap35 |
| MTJ42 | Mt Jackson | 718955 | 6650778 | Hap33 |
| MTJ43 | Mt Jackson | 718955 | 6650778 | Hap36 |
| MTJ44 | Mt Jackson | 718955 | 6650778 | Hap33 |
| MTJ52 | Mt Jackson | 719998 | 6650682 | Hap35 |
| MTJ53 | Mt Jackson | 720019 | 6650557 | Hap34 |
| MTJ54 | Mt Jackson | 720019 | 6650557 | Hap37 |
| MTJ6 | Mt Jackson | 718973 | 6650726 | Hap33 |
| MTJ7 | Mt Jackson | 718973 | 6650726 | Hap34 |
| **WAM_T98784** | Windarling | 723016 | 6677816 | Hap46 |
| **WAM_T98787** | Windarling | 723016 | 6677816 | Hap47 |
| **WAM_T98791** | Windarling | 723016 | 6677816 | Hap47 |
| **WAM_T98792** | Windarling | 723016 | 6677816 | Hap47 |
| **WAM_T98793** | Windarling | 723016 | 6677816 | Hap47 |
| **WAM_T98794** | Windarling | 723261 | 6677891 | Hap47 |
| **WAM_T98796** | Windarling | 723261 | 6677891 | na |
| **WAM_T98798** | Windarling | 723261 | 6677891 | Hap47 |
| **WAM_T98799** | Windarling | 723261 | 6677891 | Hap47 |
| **WAM_T98800** | Windarling | 723261 | 6677891 | Hap47 |
| **WAM_T98801** | Windarling | 723178 | 6677740 | Hap47 |
| **WAM_T98803** | Windarling | 723178 | 6677740 | Hap47 |
| **WAM_T98807** | Windarling | 723178 | 6677740 | Hap48 |
| **WAM_T98810** | Windarling | 723178 | 6677740 | Hap47 |
| **WAM_T98805** | Koolyanobbing | 748961 | 6581863 | Hap1 |
| **WAM_T98806** | Koolyanobbing | 748968 | 6581893 | Hap2 |
| **WAM_T98808** | Koolyanobbing | 748968 | 6581893 | Hap3 |
| **WAM_T98814** | Koolyanobbing | 749410 | 6581553 | na |
| **WAM_T98816** | Koolyanobbing | 749410 | 6581553 | Hap3 |
| **WAM_T98819** | Koolyanobbing | 749409 | 6581486 | Hap4 |
| **WAM_T98821** | Koolyanobbing | 749409 | 6581486 | Hap5 |
| **WAM_T98822** | Koolyanobbing | 749409 | 6581486 | Hap6 |
| **WAM_T98824** | Koolyanobbing | 749741 | 6581383 | Hap7 |
| **WAM_T98825** | Koolyanobbing | 749741 | 6581383 | Hap8 |
| **WAM_T98831** | Koolyanobbing | 749884 | 6581470 | Hap9 |
| **WAM_T98832** | Koolyanobbing | 749884 | 6581470 | Hap8 |
| **WAM_T98834** | Koolyanobbing | 751727 | 6580142 | Hap10 |
| **WAM_T98836** | Koolyanobbing | 751727 | 6580142 | Hap11 |
| **WAM_T98838** | Koolyanobbing | 750267 | 6579600 | Hap12 |
| **WAM_T98839** | Koolyanobbing | 750267 | 6579600 | Hap12 |
| **WAM_T98842** | Koolyanobbing | 746153 | 6583769 | Hap13 |
| **WAM_T98843** | Koolyanobbing | 744997 | 6584012 | Hap14 |
| **WAM_T98844** | Koolyanobbing | 744997 | 6584012 | Hap15 |
| **WAM_T98845** | Koolyanobbing | 744997 | 6584012 | Hap16 |
| **WAM_T98848** | Koolyanobbing | 743158 | 6585319 | Hap17 |
| **WAM_T98849** | Koolyanobbing | 743158 | 6585319 | Hap18 |
