# Supplementary material for: Strong Phylogeographic Structure in a Millipede Indicates Pleistocene Vicariance between Populations on Banded Iron Formations in Semi-Arid Australia
Source: PLoS One. 2014 Mar 24;9(3):e93038. doi: 10.1371/journal.pone.0093038 (PMC3963978; doi:10.1371/journal.pone.0093038)
Supplement: Table S2 — The three, best-fit models determined in IMa2 for tests of migration versus isolation between the paraphyletic populations Koolyanobbing and Helena Aurora. Results are based on mtDNA variation. (DOCX) [file pone.0093038.s002.docx]

| Model | log(p) | terms | df | 2LLR | AIC | Delta AIC |
| --- | --- | --- | --- | --- | --- | --- |
| Population size KOO equal to population size HA, migration rates zero | -6.091 | 2 | 3 | 1.44 | 16.182 | 0 |
| Migration rates zero | -5.371 | 3 | 2 | 0 | 16.742 | 0.56 |
| Population size KOO equal to population size 2 (ancestral population size), migration rates zero | -6.708 | 2 | 3 | 2.675 | 17.416 | 1.234 |
